# The brain health bundle: integrating evidence-based strategies to guide neurological prevention

**DOI:** 10.1186/s42466-026-00505-4

**Published:** 2026-06-02

**Authors:** Eva Schaeffer, Daniela Berg

**Affiliations:** Department of Neurology, Christian-Albrechts-University / University Hospital Schleswig-Holstein, Kiel, Germany

Neurological disorders are increasing worldwide and represent one of the most urgent challenges for modern health systems. Recent analyses from the *Global Burden of Disease 2021* study underscore the magnitude of this trend [1]. In the United States alone, an estimated 180.3 million of 332.7 million people — more than half of the population — are affected by disorders of the nervous system. With 16.6 million years lived with disability, neurological diseases constitute the leading cause of disability burden. These findings are emblematic of a global development that affects both high-income and low- and middle-income countries.

Most neurological conditions are strongly influenced by modifiable risk and progression factors. Many preventive strategies, particularly those related to lifestyle, need to be initiated early in life, as brain health is shaped by cumulative exposures from childhood through old age. At the same time, environmental challenges such as climate change, air pollution, exposure to neurotoxic substances including pesticides, and broader ecological degradation are increasingly recognized as contributors to the burden of neurological disease, not only in terms of prevalence but also with respect to more severe disease trajectories. In this sense, there can be no healthy brains in an unhealthy world.

The growing recognition of the influence of lifestyle and environmental determinants must translate into a stronger commitment to prevention. Already in 2020, the World Health Organization emphasized the need for global action to improve neurological health. Since then, major professional bodies such as the World Federation of Neurology, the European Academy of Neurology, and numerous national neurological societies have placed “brain health” and prevention at the center of their strategic agendas. This reflects a paradigm shift: from a predominantly treatment-focused approach to one that recognizes prevention as a cornerstone of neurological care. Importantly, a focus on prevention naturally implies that effective and evidence-based pharmacological, interventional and surgical prevention strategies should be integrated with non-pharmacological, lifestyle- and environment-oriented approaches to maximize their synergistic potential.

However, the concept of prevention must be specified for individual neurological disorders and grounded in robust scientific evidence. Preventive strategies, including primordial, primary, secondary, tertiary and quartiary prevention, require a clear understanding of risk factors, disease mechanisms, and effective interventions. The German Society of Neurology has therefore taken on the task of systematically compiling and critically evaluating the available evidence for prevention across major neurological disease entities in close collaboration with its clinical commissions. The aim is twofold: first, to provide a scientific foundation for the implementation of preventive measures within national health systems, with implications for health policy and economic planning; and second, to identify existing research gaps that must be addressed to improve future preventive strategies.

This special issue, referred to as the *Brain Health Bundle*, aims to support the neurological community in translating evidence into clinical practice, covering the major neurological disease areas, including dementia, epilepsy, neurovascular diseases, neuroimmunology, movement disorders, peripheral neurology, neuro-oncology, and neuroinfectiology.

*Reetz et al.* [2] highlight the profound socio-economic relevance of dementia prevention, with an estimated 129 million affected individuals worldwide by 2050, underscoring its status as a major public health priority across all levels of prevention. A set of 14 modifiable risk factors may account for up to 45% of preventable cases. Current strategies focus on multidomain interventions to enhance cognitive reserve, while emerging antibody therapies may represent secondary preventive approaches. However, major gaps persist, including limited implementation in clinical practice and insufficient structural and political support.

In their review on neurovascular diseases, *Thielscher et al.* [3] underline the substantial global burden of stroke, amounting to approximately 160 million disability-adjusted life years (DALYs). Around 23 modifiable risk factors account for over 80% of cases, including metabolic, behavioral, and environmental factors such as air pollution. Despite existing prevention initiatives, disparities in access remain, particularly in socioeconomically disadvantaged populations. Future approaches will likely include individualized risk stratification as well as digital and AI-based tools. 

*Schaeffer et al.* [4] highlight the critical role of primary prevention in shaping the future prevalence of Parkinson’s disease, both from an empirical perspective and with regard to future personalized approaches, for example in the context of gene–environment interactions. Moreover, the rapid development of early detection strategies offers largely untapped opportunities for secondary prevention in the prodromal phase. While rehabilitative strategies of tertiary prevention are already well established in clinical practice, substantial gaps remain, again particularly in lifestyle-based preventive interventions, including those targeting cognitive function in Parkinson’s disease.

In their review on neuroimmunological diseases, *Riemann-Lorenz et al.* [5] emphasize that, as in Parkinson’s disease, modifiable lifestyle and environmental factors are highly relevant across all stages of prevention. Central preventive strategies include regular physical activity, not only for risk reduction but particularly in secondary and tertiary prevention, complemented by rehabilitative approaches that require individualization and careful consideration of disease-related limitations. A healthy diet is increasingly recognized as an additional relevant factor, while vitamin D insufficiency should be corrected without promoting excessive supplementation. Alongside vitamin D, smoking remains a key modifiable risk factor in both primary and secondary prevention and should be consistently addressed in patient education. Important future goals for prevention research include a stronger focus on older populations with multiple sclerosis, taking age-related changes of the immune system into account.

As another highly prevalent condition affecting up to 30% of older adults, polyneuropathies are frequently influenced by modifiable risk factors. In their review, *Baum et al.* [6] summarize the evidence on key contributors, including B-vitamin deficiencies, diabetes mellitus, and obesity. The role of malnutrition is particularly emphasized: even in the absence of overt clinical signs, insufficient intake of essential food groups may lead to clinically relevant micronutrient deficiencies. Despite the high prevalence and clinical relevance of polyneuropathies, the authors conclude that awareness of preventive aspects remains limited and that important research gaps persist, most notably the scarcity of randomized controlled trials to guide evidence-based prevention.

For neuroinfectiology, *Klein et al.* [7] describe vaccination as a major success story in reducing disease burden of meningitis since the 1990s. However, there remains room for improvement. Prevention of pneumococcal meningitis could be strengthened by addressing emerging strains not covered by current vaccines. Varicella-zoster virus (VZV) infections have effects beyond acute disease, with studies suggesting links to neurodegeneration and dementia, underscoring the need for vaccination and preventive strategies. Recent measles outbreaks in the United Started further highlight the importance of re-strengthening vaccination programs.

How pharmacological and non-pharmacological preventive measures can form an optimal synergy is clearly illustrated in the article by *Mann et al.* on SUDEP (sudden unexpected death in epilepsy) [8]. Pharmacologically or surgically modifiable risk factors, such as frequent bilateral tonic–clonic seizures and drug-resistant epilepsy, as well as social risk factors, such as living alone, may amplify one another and therefore need to be addressed in parallel. Patient and caregiver education, along with optimization of therapy and adherence, are central components of effective prevention.

Finally, *Ippen et al.* [9]show for neuro-oncology that combined pharmacological, surgical, and radiotherapeutic strategies contribute to secondary and tertiary prevention of metastases, aiming to delay or prevent metastatic spread and disease recurrence after initial therapy. Despite advances, prevention remains underrepresented in clinical trials, highlighting the need for a stronger focus on secondary preventive approaches in future research.

Taken together, the *Brain Health Bundle* illustrates the synergy between pharmacological, interventional and non-pharmacological prevention in neurology. While some preventive factors are disease-specific, overarching elements such as healthy nutrition and physical activity are consistently relevant across conditions, highlighting the broad potential of brain health when effectively implemented. Thus, many of the included reviews emphasize the importance of behavioral prevention, including comprehensive education of healthcare professionals and patients about modifiable risk factors, alongside sustained motivation. At the same time, structural prevention is crucial to overcome the intention–behavior gap by facilitating access to preventive behaviors and reducing social inequalities. Systemic drivers of unhealthy lifestyles must be addressed at the policy level. Over recent decades, global patterns such as the widespread availability of highly processed foods, sugar-sweetened and alcoholic beverages, and sedentary behavior have contributed to increasing disease burden. A recent article in the *New England Journal of Medicine* clearly emphasized that identifiable health-harming products are key drivers of non-communicable diseases, including neurological disorders [10]. These developments illustrate how risk factors can spread across populations, underscoring the need for equally global, coordinated, and context-adapted preventive strategies involving clinicians, researchers, policymakers, and society.

## Data Availability

No datasets were generated or analysed during the current study.
